# The cost-effectiveness of deep brain stimulation in combination with best medical therapy, versus best medical therapy alone, in advanced Parkinson’s disease

**DOI:** 10.1007/s00415-013-7148-z

**Published:** 2013-10-25

**Authors:** Simon Eggington, Francesc Valldeoriola, K. Ray Chaudhuri, Keyoumars Ashkan, Elena Annoni, Günther Deuschl

**Affiliations:** 1 Medtronic International Trading Sàrl, Route du Molliau 31, 1131 Tolochenaz, Switzerland; 2 Movement Disorders Unit, Institut de Neurociències, Hospital Clínic, Universitat de Barcelona, Barcelona, Spain; 3 National Parkinson Foundation Centre of Excellence, King’s College Hospital, London, UK; 4 King’s College Hospital, London, UK; 5 Christian-Albrechts University, Kiel, Germany

**Keywords:** Best medical treatment, Cost-effectiveness, Deep brain stimulation, Parkinson’s disease, Quality of life

## Abstract

Parkinson’s disease (PD) is a complex progressive movement disorder leading to motor and non-motor symptoms that become increasingly debilitating as the disease advances, considerably reducing quality of life. Advanced treatment options include deep brain stimulation (DBS). While clinical effectiveness of DBS has been demonstrated in a number of randomised controlled trials (RCT), evidence on cost-effectiveness is limited. The cost-effectiveness of DBS combined with BMT, versus BMT alone, was evaluated from a UK payer perspective. Individual patient-level data on the effect of DBS on PD symptom progression from a large 6-month RCT were used to develop a Markov model representing clinical progression and capture treatment effect and costs. A 5-year time horizon was used, and an incremental cost-effectiveness ratio (ICER) was calculated in terms of cost per quality-adjusted life-years (QALY) and uncertainty assessed in deterministic sensitivity analyses. Total discounted costs in the DBS and BMT groups over 5 years were £68,970 and £48,243, respectively, with QALYs of 2.21 and 1.21, giving an incremental cost-effectiveness ratio of £20,678 per QALY gained. Utility weights in each health state and costs of on-going medication appear to be the key drivers of uncertainty in the model. The results suggest that DBS is a cost-effective intervention in patients with advanced PD who are eligible for surgery, providing good value for money to health care payers.

## Introduction

Parkinson’s disease (PD) is a progressive degenerative brain disorder of adult onset (average age 59 years [1]) that is estimated to affect between 0.129 and 0.151 % of the general population in Europe [2–5]. The disease is characterised by motor and non-motor symptoms; as PD advances into the complicated and late stages, motor symptoms increase in severity and frequency, freezing of gait can occur and medication-induced motor complications such as unpredictable fluctuations in symptoms and dyskinesias emerge. It is estimated that 40 % of patients experience these complications after the first 5 years from PD diagnosis, despite best medical therapy (BMT) [6].

Parkinson’s disease of all severities can have a significant impact upon patients’ quality of life (QoL) [7–12], mainly due to increased disability, pain, motor complications and falls [13–25]. As the disease progresses, the mental, physical, social and emotional domains of patients’ QoL decrease significantly [26]. High costs are incurred during the course of PD in terms of drug therapy, hospitalisations and fall-related injuries; European studies have estimated that the total cost per PD patient is €10,000–14,000 per year, of which approximately €6,000 is due to lost productivity [27, 28]. Costs increase significantly with disease progression [27, 29].

The management of patients with PD varies according to disease stage and consists mainly of drug therapy. For advanced disease, the European Federation of Neurological Societies (EFNS) recommends various oral drug therapies as monotherapy or combination therapy [30]. As patients become refractory to oral drugs despite BMT, deep brain stimulation (DBS) has been shown to be an effective therapy in combination with BMT, versus BMT alone [31–36]. In the UK, current guidance from the National Institute for Health and Clinical Excellence (NICE) recommends the use of DBS for patients who have motor complications and who are refractory to medical treatment [37, 38].

The majority of existing cost-effectiveness analyses [39–42] have limitations as they report short-term results only [39], represent health states using non-standard measures such as nursing home status as opposed to formal disease modelling [40], or have not carried out a formal cost-effectiveness analysis [41]; no analysis has been based on patient-level randomised controlled trial (RCT) data or has used the UK NHS perspective. Our study objective was to develop a new cost-effectiveness model to compare interventions in advanced PD, based on patient-level RCT data using longer-term costs and disease outcomes. Specifically, our analysis sought to estimate the cost-utility of DBS in combination with BMT, compared with BMT alone in patients with advanced PD, from a UK payer perspective.

## Methods

### Overview of model

We developed a Markov model using patient-level data from the 6-month RCT by Deuschl et al. [31], which compared DBS plus BMT vs. BMT alone in 156 patients with advanced PD. The study evaluated changes in quality of life via the Parkinson’s disease questionnaire (PDQ-39), and changes in symptom severity using Part III of the UPDRS. The study also recorded H&Y stage at each visit (ranging from 0 to 5, 0 representing a patient free of PD symptoms and stage 5 representing a wheelchair-bound patient requiring constant nursing care) [43], level of ‘OFF’ time (proportion of the waking day in which the patient’s medication is not providing adequate symptom control) and a series of demographic parameters.

**Fig. 1:**
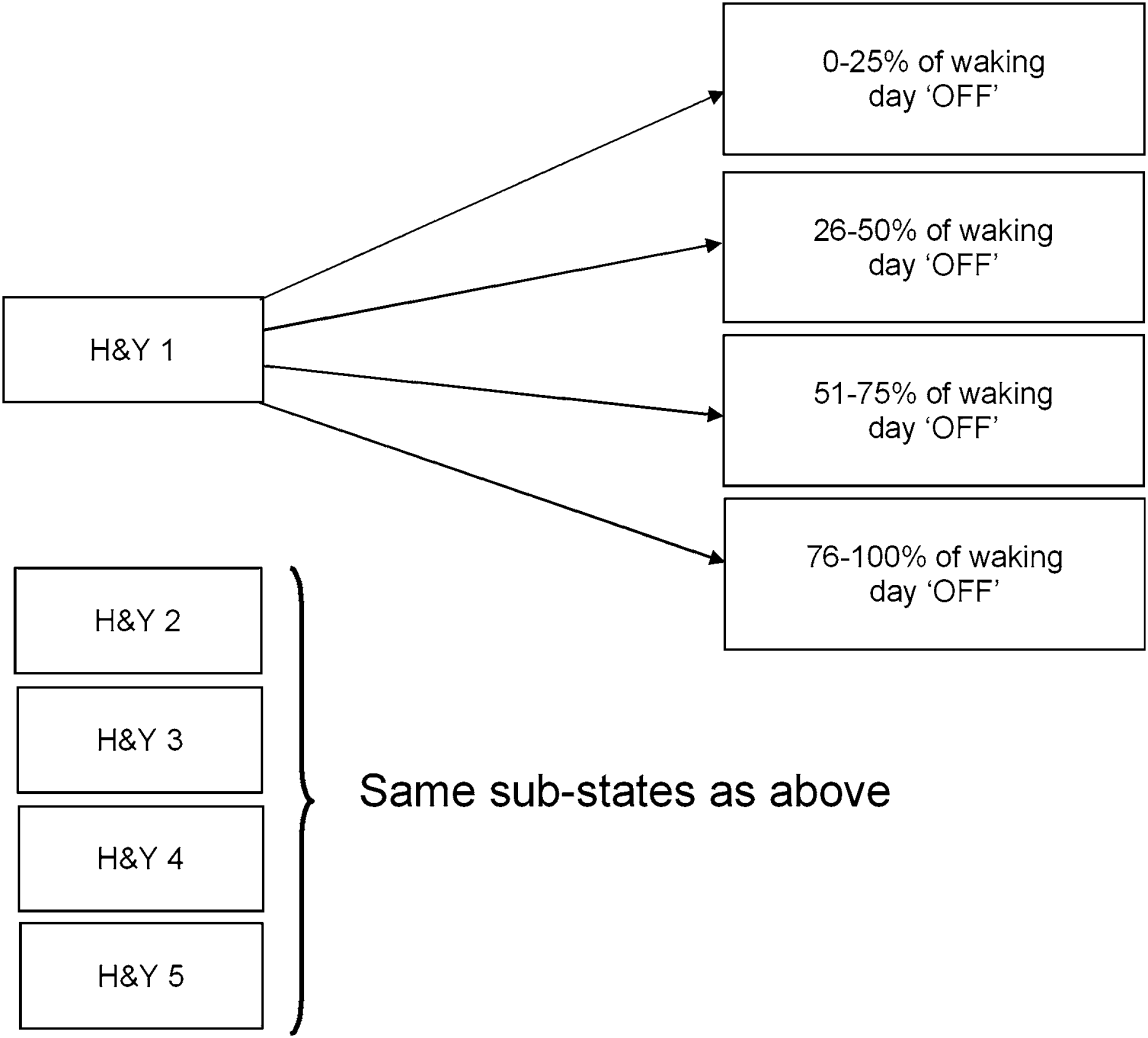
Model health states (H&Y and ‘OFF’ time). The H&Y scale focuses on motor symptoms and classifies patients into one of six categories (ranging from 0 to 5) according to disease severity, with 0 representing a patient free of PD symptoms and stage 5 representing a wheelchair-bound patient requiring constant nursing care. The amount of time a patient spends in the ‘OFF’ state per day is an aspect of the UPDRS, which assesses various aspects of the disease, including mental status, motor function and complications of therapy, and assigns a total score to each patient reflecting an overall estimate of the degree of disability

The model defines health states according to the H&Y stages (1–5), with each state split into four sub-states to represent the level of ‘OFF’ time (see Fig. 1). In addition, death was included as an absorbing state. A Markov approach was deemed suitable given the logical separation of patients into these various disease stages. A 6-month cycle length was used, given that the available trial data consisted of a baseline and 6-month visit, together with a 5-year time horizon to reflect the uncertainty in long-term outcomes while also capturing the costs associated with device replacement. A UK NHS perspective on costs was adopted, with both costs and quality-adjusted life-years (QALYs) discounted at 3.5 % per annum, in line with recommendations from NICE [44].

In each cycle, patients could worsen by one H&Y stage, progress by one level of ‘OFF’ time, or both. If none of these events occurred, the patient remained in the same health state in the next model cycle. The model assumed that, after the first 6 months of treatment, patients’ H&Y stage or ‘OFF’ time cannot be improved further. Any patient withdrawing from treatment in the DBS arm was assumed to immediately progress to the next worst ‘OFF’ time state, to reflect the expected reduction in symptom control when the device is switched off or explanted. Table 1 summarises the key model input parameters.

**Table 1:** Model input parameters and values

Parameters	Value	Source/comment
6-month probability of progression		
H&Y 1 to 2	0.188	Zhao et al. [45]
H&Y 2 to 3	0.04	Zhao et al. [45]
H&Y 3 to 4	0.159	Zhao et al. [45]
H&Y 4 to 5	0.148	Zhao et al. [45]
6-month probability of progression		
0–25 to 26–50 % ‘OFF’ time	0.127	Palmer et al. [46, 47]
26–50 to 51–75 % ‘OFF’ time	0.074	Palmer et al. [46, 47]
51–75 to 76–100 % ‘OFF’ time	0.043	Palmer et al. [46, 47]
Utilities in H&Y 1		
0–25 % OFF time	0.74	Palmer et al. [46]
26–50 % OFF time	0.68	Palmer et al. [46]
51–75 % OFF time	0.64	Palmer et al. [46]
76–100 % OFF time	0.52	Palmer et al. [46]
Utilities in H&Y 2		
0–25 % OFF time	0.72	Palmer et al. [46]
26–50 % OFF time	0.72	Palmer et al. [46]
51–75 % OFF time	0.66	Palmer et al. [46]
76–100 % OFF time	0.49	Palmer et al. [46]
Utilities in H&Y 3		
0–25 % OFF time	0.643	Lowin et al. [48]
26–50 % OFF time	0.555	Lowin et al. [48]
51–75 % OFF time	0.467	Lowin et al. [48]
76–100 % OFF time	0.379	Lowin et al. [48]
Utilities in H&Y 4		
0–25 % OFF time	0.387	Lowin et al. [48]
26–50 % OFF time	0.299	Lowin et al. [48]
51–75 % OFF time	0.211	Lowin et al. [48]
76–100 % OFF time	0.123	Lowin et al. [48]
Utilities in H&Y 5		
0–25 % OFF time	0.131	Lowin et al. [48]
26–50 % OFF time	0.043	Lowin et al. [48]
51–75 % OFF time	−0.045	Lowin et al. [48]
76–100 % OFF time	−0.133	Lowin et al. [48]
Relative risks of mortality		
H&Y 2 (vs. H&Y 1)	2.03	Liou et al. [49]
H&Y 3 (vs. H&Y 1)	2.16	Liou et al. [49]
H&Y 4 (vs. H&Y 1)	4.99	Liou et al. [49]
H&Y 5 (vs. H&Y 1)	4.99	Liou et al. [49]
Drug dosing		
Daily levodopa dose (mg) in BMT arm (first cycle)	220	Deuschl et al. [31]
Daily levodopa dose (mg) in BMT arm (subsequent cycles)	205	Deuschl et al. [31]
Daily levodopa dose (mg) in DBS arm (first cycle)	213	Deuschl et al. [31]
Daily levodopa dose (mg) in DBS arm (subsequent cycles)	188	Deuschl et al. [31]
Adverse events—DBS (per cycle)		
System infections per patient during first cycle (12 out of 121 patients had a total of 16 infections)	0.132	Weaver et al. [32]
Probability of DBS infection in subsequent cycles	0.026	Deuschl et al. [31]
Probability per cycle of lead dislodgement	0.066	Weaver et al. [32]
Probability of withdrawal from DBS during first cycle	0.1	Deuschl et al. [31]
Probability of withdrawal from DBS during each subsequent cycle	0.02	Assumption
Adverse events—both treatment arms		
Number of falls per cycle for patients with H&Y 3	3.15	Pickering et al. [50]
Relative risk of fall for patient with H&Y 4 or 5 (vs. H&Y 3)	1.72	Pickering et al. [50]
Probability of hospitalisation per fall	0.62	Bloem et al. [51]
Treatment withdrawal rates		
Probability of withdrawal from DBS during first cycle	0.1	Deuschl et al. [31]
Probability of withdrawal from DBS during each subsequent cycle	0.02	Assumption
Cost parameters—drug acquisition		
Cost per cycle of drugs in BMT arm (excluding levodopa) or for DBS patients who’ve withdrawn	£3,725	McIntosh et al. [52]
Cost per cycle of drugs in DBS arm (excluding levodopa)	£2,109	McIntosh et al. [52]
Cost per levodopa tablet (100 mg levodopa/10 mg Carbidopa)	£0.07	British National Formulary No. 62 (2011) [53]
Cost parameters—treatment initiation, materials and implantation		
Pre-operative assessment/work-up	£641	Payment by results tariffs (2011–2012) code: AA25Z [54]
DBS device	£8,326	Medtronic UK price list. Activa IPG, model number 37,601 [55]
DBS extensions (cost of 2 extensions)	£1,530	Medtronic UK price list. Stretch coil extension, model numbers 3,708,540, 3,708,560, 3,708,595 [55]
DBS leads (cost of 2 leads)	£1,786	Medtronic UK price list. Lead kit, model numbers 338,728, 338,740 [55]
DBS patient programmer	£560	Medtronic UK price list. Activa patient programmer, model numbers 37,642 [55]
DBS implantation procedure	£7,131	Payment by results tariffs (2011–2012) Code: AA072Z [54]
Cost parameters—adverse event management		
Infection (DBS)	£10,690	Payment by results tariffs (2011–2012) codes: AA04Z and PA18B [54]. Plus costs of new device, leads and extensions for serious infections.
Lead dislodgement (DBS)	£8,789	Repeat implantation procedure, new leads, new extensions
System explantation (DBS)	£6,976	Payment by results tariffs (2011–2012) code: AA07Z [54]
Battery replacement procedure (DBS)	£616	Payment by results tariffs (2011–2012) code: DZ06Z [54]
Withdrawal from DBS (device switched off)	£217	Payment by results tariffs (2011–2012) code: 400 (non-mandatory) [54]
Hospitalisation due to fall (all arms)	£294	Payment by results tariffs (2011–2012) code: WA23X [54]
Cost parameters—follow-up		
Cost per neurosurgery follow-up visit (1 visit in first cycle post-implantation of DBS)	£283	Payment by results tariffs (2011–2012) code: 150 (non-mandatory) [54]
Cost per outpatient neurology appointment (3 visits per 6 months assumed in all arms)	£217	Payment by results tariffs (2011–2012) code: 400 (non-mandatory) [54]
Cost per PD nurse visit (3 home visits assumed per 6 months in all arms)	£114	Curtis [56]

*DBS* deep brain stimulation, *H&Y* Hoehn and Yahr, *PD* Parkinson’s disease

### Patient-level efficacy data

**Fig. 2:**
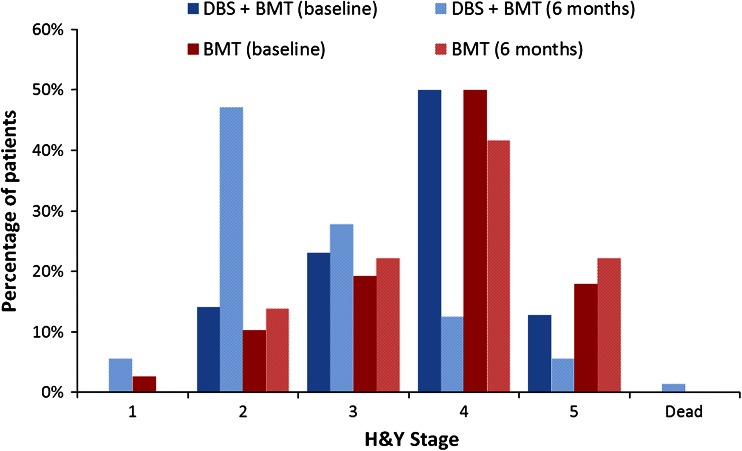
Hoehn and Yahr stage at baseline and 6 months [31]

**Fig. 3:**
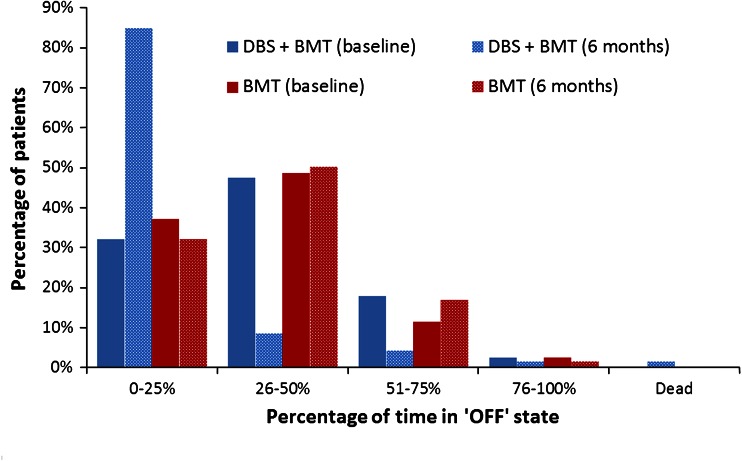
‘OFF’ time at baseline and 6 months [31]

The trial data comprised baseline and 6-month data on H&Y stage and level of ‘OFF’ time for each patient, enabling us to estimate the state populations at these two time points. Figure 2 shows the split of patients between the H&Y stages in each arm at baseline and 6 months. Since our model also addresses the amount of ‘OFF’ time per patient, we also compared the two arms on this outcome at baseline and 6 months (Fig. 3).

We used the data from Figs. 2 and 3 to represent the changes in patients’ H&Y stage and level of ‘OFF’ time between baseline and 6 months, and subsequently modelled long-term progression of PD in two ways. Although long-term studies of DBS in PD patients do exist, the data available from these studies was not sufficiently detailed to allow disease progression probabilities to be estimated between the health states in the model. We therefore modelled progression of patients’ underlying disease stage using data reported between the various H&Y stages [45]. Using the time-to-event curves reported for each progression event (e.g. H&Y 1–H&Y 2, H&Y 2–H&Y 3), we calculated 6-month probabilities of progression between each successive H&Y stage and applied these to both arms of the model from 6 months onwards. We also modelled worsening of ‘OFF’ time using data from previous economic evaluations [46, 47], again applying these probabilities equally to both treatment arms.

### Mortality

The trial data reported that a total of four patients died during the 6-month follow-up period (three in the DBS group, and one in the BMT group) [31]. Of the deaths in the DBS group, one was due to a cerebral haematoma, one patient committed suicide, and one died of pneumonia; in the BMT group, the single death was due to a car accident. With the exception of the cerebral haematoma, none of these deaths were considered to be related to the interventions received (recent evidence has shown no association between DBS and an increased risk of suicide [57]) and so the remaining three deaths were excluded from the mortality calculations in the model.

In order to capture the effect of increased mortality risk for individuals with PD and the relationship between disease status and mortality, we used age-specific all-cause general population mortality rates from the UK [58], to which we applied relative risks within each H&Y stage to reflect increasing mortality risk with disease progression, using data from a community-based study [49].

### Quality of life

To capture the effects of PD progression upon patients’ QoL, we applied utility weights to each of the health states in the model. Although PDQ-39 data were collected at both visits within the key clinical study [31], there is currently no adequate tool for mapping these data to a generic measure of QoL suitable for use in decision-analytic modelling e.g., EQ-5D. For this reason, we used utility data from two previous economic evaluations [46, 48], which provided utility estimates covering all of the health states included in our model (i.e., for each combination of H&Y and ‘OFF’ time). The modelled benefit of DBS was therefore based on the distribution of patients between health states with different utilities, rather than through separate modelling of treatment-specific utilities.

### Resource use and costs

The model included the relevant costs relating to each treatment arm: device acquisition and implantation; adverse event management (infection, lead dislodgement, battery exhaustion, hospitalisation for falls); PD nurse home visits (three per cycle); drug therapy (levodopa and other anti-Parkinson medication); routine follow-up (three neurology outpatient visits per cycle). The DBS arm was associated with the up-front costs of the device and its implantation, including a pre-operative assessment, to which were added the costs of device-related complications (infections and lead displacements). The majority of infections occur at the site of the pulse generator soon after implantation (12 patients out of 121 had a total of 16 infections over 6 months, thus a rate of 0.132 per patient was assumed) [32] and are relatively more easily managed, while in other cases the infection spreads to the leads situated in the brain, requiring a full explantation of the system. Our analysis conservatively assumed that half of infections are of the severe type requiring system explantation, with the cost applied according to this weighting. The baseline rate of falls was assumed to be 3.15 per cycle for patients in H&Y 3 [50]; no falls were assumed in H&Y 1 or 2, and relative risks were applied to the baseline rate to reflect higher fall rates in H&Y 4; 5.60 % of falls were assumed to result in hospitalisation [51].

Drug costs were estimated via levodopa use data from the key clinical study [31], together with data reported on drug therapy costs from a separate DBS study [52]. For device replacement, which is assumed to occur at 4 years (based on the Medtronic Activa^®^ PC DBS device), we applied the cost of a new device plus the procedure cost for making the replacement. In addition, the costs associated with any new infections arising from these replacements were included. Follow-up costs in both arms were based on the assumption of three neurology outpatient appointments and three PD nurse visits per cycle, which would cover general follow-up, including adjustment of the DBS stimulation parameters. These assumptions were validated with clinical experts and were applied equally to both arms of the model. A per-cycle probability of withdrawal from DBS was applied, after which on-going management costs for these patients were assumed to be equivalent to patients in the BMT arm. The cost of management of cerebral haematoma in the DBS group was excluded from the cost analysis, since the death observed in the clinical study occurred during the procedure and would therefore be covered by the tariff paid to the hospital for the DBS implantation procedure.

The cost year used was 2011. Price uplifts were used where necessary to inflate costs to current values.

### Cost-utility analysis

The primary outcome of interest from cost-effectiveness analyses and from this model is the incremental cost-effectiveness ratio (ICER), which describes the additional cost per health unit gained. In this model, we calculated the cost-utility in terms of the cost per QALY gained. The ICER is calculated as follows for the treatment comparison being made in this study:

$${\text{ICER}} = \frac{{C_{\text{DBS}} - C_{\text{BMT}} }}{{Q_{\text{DBS}} - Q_{\text{BMT}} }}$$

where *C*_DBS_ and *C*_BMT_ represent the total costs associated with the DBS and BMT groups, respectively, and *Q*_DBS_ and *Q*_BMT_ represent the total QALYs for each intervention.

### Sensitivity analysis

In order to explore the impact of parameter uncertainty upon the cost-effectiveness results, a series of one-way sensitivity analyses was undertaken to identify key parameters in the model. The results can then be presented in the form of a tornado diagram, which shows the relative importance of each input parameter in terms of the effect upon the ICER of using smaller and large values of each input. The range chosen for each input value was based either on the published confidence interval or upon plausible alternative values from the literature. For example, two different scenarios for the utility weight parameters were explored, using utilities reported from two alternative quality of life studies [59, 60].

## Results

### Base-case analysis

Table 2 shows the discounted results of the base-case cost-effectiveness analysis over a 5-year time horizon. These results give an incremental cost-effectiveness ratio (ICER) of £20,678 per QALY gained. The incremental costs in the DBS arm were largely made up of the costs of the device and its implantation, although some of these costs were offset by reduced drug use (both levodopa and other anti-Parkinson medication) and fewer falls. The initial clinical benefit observed in the DBS arm of the key clinical study [31] (in terms of improved H&Y stage and reduced ‘OFF’ time) was projected to translate into a substantial QALY gain for these patients over the 5-year period. The model predicted a modest survival gain for patients in the DBS arm (approximately 1 month on average over the model horizon), which was observed due to the increasing risk of death applied for patients in H&Y 4 and 5 (the 5-year mortality rates predicted by the model were 13.7 and 17.2 % for the DBS and BMT arms, respectively).

**Table 2:** Base-case cost-effectiveness results

Treatment	Costs	QALYs
BMT	£48,243	1.21
DBS + BMT	£68,970	2.21
Incremental	£20,727	1.002

*BMT* best medical treatment, *DBS* deep brain stimulation, *QALY* quality adjusted life year

### Sensitivity analysis

**Fig. 4:**
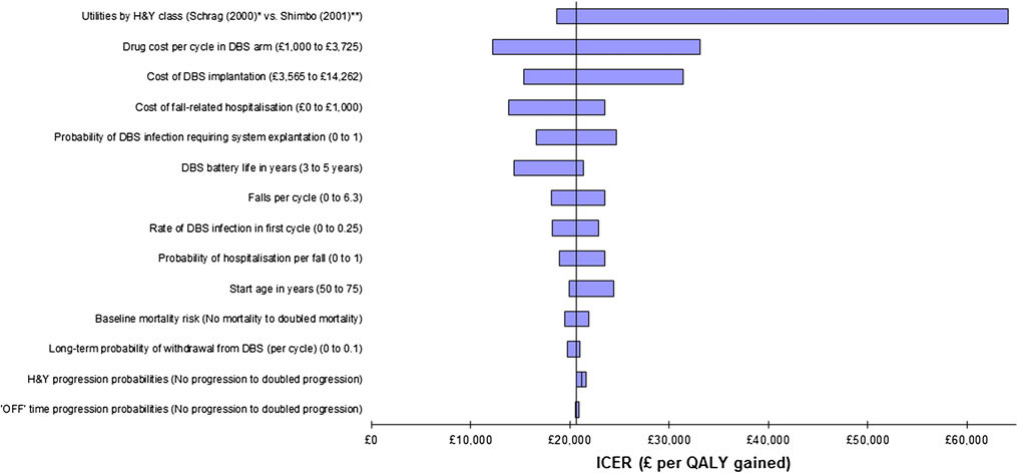
* Schrag et al. [60] reported utilities by H&Y class: 0.96 (H&Y 1); 0.65 (H&Y 2); 0.26 (H&Y 3); 0.19 (H&Y 4); −0.21 (H&Y 5). There is a greater discrepancy between the H&Y stages in this study than in the base-case analysis. ** Shimbo et al. [59] utilities: 0.708 (H&Y 1); 0.678 (H&Y 2); 0.622 (H&Y 3); 0.547 (H&Y 4); 0.451 (H&Y 5). There is less discrepancy between the H&Y stages in this study than in the base-case analysis. In both of these scenarios, the H&Y utilities were applied across all levels of ‘OFF’ time. The most influential parameters are shown at the top of the chart

Figure 4 shows the results of the one-way sensitivity analysis in the form of a tornado diagram. The most influential parameters, in terms of their effect upon the ICER, appear at the top of the chart (those with the widest bars).

There was minimal effect upon the ICER for many of the model parameters. The key inputs were the utility weights applied to each health state. When QoL was considered to vary by H&Y stage only (i.e., the effect of ‘OFF’ time on QoL is ignored) and utility data from a different economic evaluation used [59], the ICER increased to £64,170 (the extreme right of the top bar in the chart)—this result differed greatly from the base-case result because of the greater similarity of utility weights in each H&Y stage from that study. A separate study reported a wider spread of utility weights across the H&Y stages [60], resulting in an ICER more consistent with the base-case result (£18,650 per QALY gained—see the extreme left of the top bar in the chart). A further sensitivity analysis on the QoL data applied utility weights from a different study to the four levels of ‘OFF’ time (thus ignoring any differences in QoL between the H&Y stages)—this too caused a significant increase in the ICER because the utility weights allowed less differentiation between the health states used in the model. One further study reported separate utility weights for each level of ‘OFF’ time [61]; applying these utilities in the model also led to a higher ICER.

Varying the per-cycle drug cost in the DBS arm caused variation in the ICER, since this cost was applied in every model cycle. When the cost was set equal to the equivalent cost in the BMT arm, the ICER increased to around £33,079 per QALY gained. Similarly, the cost of the DBS implantation procedure represented a significant component of the overall cost in the DBS arm; thus, variability in this input altered the ICER. However, the procedure cost would need to be doubled in order for the ICER to go above £30,000 per QALY. The proportion of infections which require a full DBS system explantation and the cost of fall-related hospitalisations also had an impact upon the ICER, but to a lesser extent.

## Discussion

Based on this cost-effectiveness analysis, using a Markov model and patient-level data from a randomised controlled trial, DBS in combination with BMT offers value for money to UK payers for the treatment of PD, with an ICER of £20,678 per QALY gained compared to BMT alone over a 5-year period. This ICER is below the NICE cost-effectiveness threshold and compares favourably with existing interventions funded on the NHS [62, 63]. The model improves upon previous economic evaluations by capturing changes in both H&Y stage and level of ‘OFF’ time for each intervention, and used a 5-year horizon to ensure that the costs of DBS battery replacements were accounted for.

The high up-front device and surgery costs were outweighed by gains in QoL and reduced drug use. Although the model predicted minimal survival gain, the benefit of DBS was gained through improvements in H&Y stage and ‘OFF’ time, which led to gains in quality of life and thus quality-adjusted survival. The key aspect of the model was the initial benefit of DBS, and the sensitivity analyses have demonstrated that the results are sensitive to changes in the QoL inputs and the costs of on-going drug therapy. Since many parameters in the model were common to both arms, the impact of changing these parameters was minimal.

There are currently few economic evaluations of DBS against which to compare the results of this study. One study reported an ICER of €34,389 per QALY gained for DBS versus BMT using a 1-year time horizon, based on a longitudinal study of patients with advanced PD [39], a result which was largely driven by significant QoL gains for patients on DBS; the short time horizon used may explain the higher ICER derived from the analysis than in the results presented here. A second cost-effectiveness analysis comparing DBS and BMT reported an ICER of $49,194 per QALY gained over a lifetime horizon, though this study used Markov states based around nursing home residency rather than the underlying disease, and many of the input parameters were not evidence-based [40]. The ICER from our analysis is consistent with that reported by NICE (£19,500 per QALY gained) in the clinical guidelines for PD [38]; this study also used a 5-year horizon but did not formally model a disease process, instead calculating QALYs based on percentage changes in UPDRS scores. Our study is also consistent with the 5-year cost-utility (ICER €27,958 per QALY) resulting from a recent German Markov model analysis [42]. Our model used a 5-year horizon to capture longer-term costs and outcomes and, by modelling progression in terms of H&Y stage and level of ‘OFF’ time using patient-level data, offers a realistic representation of the course of the disease.

To validate the prediction of long-term outcomes for patients in the model, we compared the predicted H&Y mix for DBS patients against 2-year data reported in a previous study [32, 64]. Our model predicted a mean H&Y stage of 2.91 amongst surviving DBS patients at 2 years (from a baseline of 3.62), compared with 3.0 from the 2-year study (from a baseline value of 3.4) (Additional follow-up data from the PD SURG study will be important in further validation of our model. A comparison of the mortality rates predicted by our model against existing literature on survival for patients with PD suggests that our model may over-estimate survival times. Several clinical studies have shown significantly higher mortality rates than those predicted by the model, with 8–10-year mortality ranging from 38 to 92 % [65, 66]. Much of this difference can be accounted for by comparing the age profile of patients in these studies (mean age at baseline 70–75 years) with the baseline age of patients in our model (60.5 years, based on the key clinical study [31]). By modifying the baseline age of patients in the model to 75 years, the predicted mortality estimates correlated more closely with the clinical evidence.

A number of aspects of the analysis warrant further discussion with respect to uncertainty in long-term outcomes, transferability of data and disease status indicators. The key limitation of the study is that the clinical benefit projected for patients on DBS is based largely around short-term evidence, extrapolated to a 5-year horizon using alternative data sources. The model would benefit from trial data with a longer follow-up period, which would allow the persistence of the treatment effect to be more robustly represented. The model was not sensitive to changes in the long-term disease progression inputs, since they were applied to both arms of the model—treatment-specific long-term data would enable this to be explored in more detail. The PD SURG study should help to address this data gap [33].

Secondly, the QoL data used in the model do not come from a study of DBS, and therefore the transferability of this data is uncertain. The model assumed that QoL is related to H&Y stage and level of ‘OFF’ time, which allowed differentiation between the treatment arms in the calculation of QALYs; however, treatment-specific utility data would address this aspect of the model more appropriately. The current evidence based contains a wide range of utility weights for patients with PD, and we have evaluated the impact of these within the sensitivity analysis. Related to this, a recent review of economic modelling studies in interventions for PD noted that, of the 18 model-based evaluations assessed, none addressed the issue of non-motor symptoms (e.g., dementia, depression, sleep disorders), instead focusing entirely on motor-related outcomes [67]. Studies have shown that non-motor symptoms can have a significant impact upon patients’ QoL [11, 68, 69], and further data on the impact of treatment upon such symptoms and the associated effect upon QoL would help to fully capture this aspect of the disease. Our model has addressed this by assigning utility weights according to both H&Y stage and ‘OFF’ time, thus capturing a broader range of the disease aspects which influence QoL.

Thirdly, the model predicted that DBS provides a small survival benefit over BMT. Although evidence does exist to suggest a survival benefit for DBS patients [70], this was a single-centre study and further evidence is required to support this outcome. The sensitivity analysis undertaken, however, suggests that when the mortality risk is set to be equal across all health states, there is minimal impact on the cost-effectiveness results, so this appears to be more an issue of face validity.

Still, despite these limitations, the model captures the most important aspects of costs and effects of the addition of DBS to BMT and how they compare with those of standard BMT in the longer-term, thus providing important information on the value for money of the therapy for evidence-based decision-making. This analysis was undertaken from the UK perspective; adaptations to other countries will complement the cost-effectiveness evidence on DBS. Future analyses also need to assess the cost-effectiveness of DBS in patients with early stage disease, who have also recently shown to benefit from DBS on a range of outcomes [71, 72].

## Conclusions

This evaluation suggests that DBS may be considered a cost-effective intervention from a UK payer perspective when compared with BMT alone (ICER £20,678) in patients with advanced PD eligible for surgery, providing good value for money to payers. Parkinson’s disease is a complex condition, and this Markov model has captured the key aspects of the disease in terms of both disease and economic outcomes. Further evidence on long-term disease outcomes, including quality of life, plus head-to-head trial evidence against other comparators in this indication, would be valuable in facilitating further research on the economic aspects of these interventions. Unfortunately, the treatment outcome of pump-administered drug infusion therapies is much less established according to evidence-based medicine criteria, and authoritative economic data on these therapies are rare. Early estimates suggest higher costs for pump-administered drug infusion therapies, but future studies are needed for comparisons.
